# Gene expression studies using a miniaturized thermal cycler system on board the International Space Station

**DOI:** 10.1371/journal.pone.0205852

**Published:** 2018-10-31

**Authors:** Tessa G. Montague, Alia Almansoori, Emily J. Gleason, D. Scott Copeland, Kevin Foley, Sebastian Kraves, Ezequiel Alvarez Saavedra

**Affiliations:** 1 Department of Molecular and Cellular Biology, Harvard University, Cambridge, Massachusetts, United States of America; 2 Al Mawakeb School Al Barsha, Dubai, UAE; 3 miniPCR, Cambridge, Massachusetts, United States of America; 4 Boeing, Houston, TX, United States of America; Charles P. Darby Children's Research Institute, 173 Ashley Avenue, Charleston, SC 29425, USA, UNITED STATES

## Abstract

The distance and duration of human spaceflight missions is set to markedly increase over the coming decade as we prepare to send astronauts to Mars. However, the health impact of long-term exposure to cosmic radiation and microgravity is not fully understood. In order to identify the molecular mechanisms underpinning the effects of space travel on human health, we must develop the capacity to monitor changes in gene expression and DNA integrity in space. Here, we report successful implementation of three molecular biology procedures on board the International Space Station (ISS) using a miniaturized thermal cycler system and *C*. *elegans* as a model organism: first, DNA extraction–the initial step for any type of DNA analysis; second, reverse transcription of RNA to generate complementary DNA (cDNA); and third, the subsequent semi-quantitative PCR amplification of cDNA to analyze gene expression changes in space. These molecular procedures represent a significant expansion of the budding molecular biology capabilities of the ISS and will permit more complex analyses of space-induced genetic changes during spaceflight missions aboard the ISS and beyond.

## Introduction

Human spaceflight is set to undergo a major shift. While the last few decades have seen short- or medium-term (<1 year) orbital flights within Earth’s protective magnetic field, in the coming years astronauts will transition to longer-term explorations of deep space. There are many challenges associated with deep space missions, but the predominant health risks that must be mitigated before humans can safely undergo interplanetary travel are the consequences of exposure to cosmic radiation and microgravity. The high-energy protons and high-charge and high-energy nuclei that comprise cosmic rays can have devastating effects on human health. One of the most vulnerable sites for cosmic radiation damage is DNA, where mutations can lead to the development of cancer [1,2]. In addition, the combination of microgravity and cosmic radiation can negatively impact many normal biological processes in the skeletal, immune and nervous systems of humans [3] and other organisms [4–7]. In many cases, alterations in gene expression precede and accompany the physiological changes [8–19], providing a means to predict and understand the nature of the damage. Thus, during long-term space missions, detecting molecular changes in the DNA and RNA of astronauts will be useful to monitor their health and inform possible treatments.

Recent developments in molecular biology tools on the ISS have resulted in the first reports of DNA amplification and sequencing in space [20,21]. However, a larger repertoire of nucleic acid techniques will be needed to render the ISS capable of complete molecular biology procedures. The recent WetLab-2 mission has enabled RNA extraction and quantitative PCR to be performed on board the ISS [22], although substantial space and resources are required to use this system. To maximize space, cost and astronaut efficiency, it would be useful for astronauts to have the capacity to implement basic molecular biology procedures rapidly in space. In this Genes in Space-4 study (https://www.genesinspace.org/) we tested three of the major techniques used on Earth to monitor gene expression and detect DNA sequence changes on board the ISS using only a miniaturized thermocycler system, which has been successfully used for DNA studies on Earth [23] and in space [21]. We used the nematode worm *Caenorhabditis elegans* (*C*. *elegans)* as a model organism, which has already been successfully flown to space for scientific study [24]. We performed a DNA extraction experiment, we reverse transcribed RNA into cDNA, and performed semi-quantitative PCR on board the ISS. These experiments provide a foundation for more complex experimental procedures to monitor molecular changes to human health in space.

## Methods

### Strains

*C*. *elegans* were maintained at 20°C. N2 Bristol was used as wild-type. MT17463 [*set-25(n5021)*] animals [25] contain a 1978 bp deletion in the *set-25* gene. *set-25* mutants were genotyped using two primer pairs. The outer primers (S1 Table) produce a 2220 bp band with wild-type DNA, and a 242 bp band with *set-25* DNA; the inner primers produce a 573 bp band with wild-type DNA, and no PCR product with mutant DNA.

### DNA extraction

Either 1 or 2 animals were placed in tubes with 5 μL of lysis buffer (50 mM KCl,10 mM Tris pH 8.3, 2.5 mM MgCl_2_, 0.45% IGEPAL (Sigma, I8896), 0.45% Tween-20 and 0.01% Gelatin) supplemented with 100 μg/mL proteinase K and the tubes were frozen at -80°C. The samples remained frozen until operations on board the ISS. The lysis protocol on the miniPCR machine was: 60°C/60 min, 95°C/15 min. However, due to input error, the heat inactivation step in space was carried out for 1.5 minutes instead of 15 minutes. Thus, upon their return to Earth, the samples were subjected to the remaining 13.5-minute heat inactivation. On Earth, 1 μL of the extracted DNA was placed in tubes with OneTaq Hot Start Taq kit (NEB, M0484S) and 0.4 μM of the *set-25* primers for DNA amplification using the following protocol: 95°C/2 min, [95°C/30 sec, 55°C/30 sec, 72°C/3 min] x35, 72°C/5 min. The completed PCR reactions were run on a 2% agarose gel.

### Heat shock

Worms were synchronized and grown to 4 hours post-L4 stage on NGM agar plates and submerged in a 33°C water bath for 30 minutes. The control “no heat shock” plates were maintained at room temperature. The heat shocked plates were allowed to recover for 30 minutes at 20°C before washing the worms off the plates and freezing them in liquid nitrogen.

### RNA extraction and DNase digestion

RNA was extracted from the worms using TRIzol (ThermoFisher, 15596026). Briefly, frozen worms were resuspended in TRIzol, flash frozen in liquid nitrogen, and then thawed at 37°C and vortexed for 1 minute. The freeze-thaw-vortex cycle was repeated twice, and the samples were combined with chloroform and centrifuged at 4°C for 15 minutes before the aqueous layer was removed and combined with isopropanol. The RNA was pelleted by spinning at 4°C for 15 minutes, the pellets were washed in ethanol and then resuspended in RNase-free water and combined with Turbo DNase and buffer (ThermoFisher, AM2239). DNase digestion was carried out at 37°C for 30 minutes. An additional 1 μL of Turbo DNase was added to the reactions, they were incubated for a further 30 minutes, and then RNA was purified from the samples by ethanol precipitation.

### cDNA synthesis

For the two-step reverse transcription and PCR, 1 μg of RNA from each condition was combined with 1 μL of iScript reverse transcriptase (Bio-Rad, 1708890) and 4 μL of iScript buffer in a total reaction volume of 20 μL. The cDNA synthesis program was as follows: 25°C/5 min, 46°C/20 min, 95°C/1 min.

### Semi-quantitative PCR

For the combined one-step RT-PCR, 100 ng of RNA from each condition was added to a tube and combined with OneTaq One-Step RT-PCR mix (NEB, E5315S) and 0.4 μM of each primer (S1 Table). The reactions were frozen at -80°C and maintained in a frozen state until operations on board the ISS. The RT-PCR protocol was as follows: 48°C/20 min, [94°C/20 sec, 56°C/30 sec, 68°C/60 sec] x24/28, 68°C/10 sec. The two-step semi-quantitative PCR combined 1 μL of cDNA as the template, OneTaq Hot Start master mix (NEB, M0484S) and 0.4 μM of each primer. The samples were frozen at -80°C and remained frozen until operations on board the ISS. The PCR protocol was identical for the one-step and two-step protocols. Upon returning to earth, the samples were run on a 2% agarose gel for analysis.

### Thermal cycler system

All experiments were carried out in a mini8 miniPCR thermal cycler (www.minipcr.com).

### ISS operations

The Genes in Space samples were prepared on Earth, frozen, and sent on dry ice to the Kennedy Space Center, where they arrived 6 days before the launch. The samples were stored in a POLAR freezer until 24 hours prior to launch when they were moved to the SpaceX Dragon vehicle, kept at -95°C, and launched on a SpaceX Falcon rocket to the ISS. Upon docking with the ISS, the samples were transferred to the GLACIER -95°C freezer on orbit until further processing. During experimental operations, the ISS crew removed the samples from the freezer, allowed them to thaw, and took preliminary photos to show the sample strip conditions prior to being removed from the flight bag. A visual inspection of the samples strips was also performed at this time to verify all sample tube caps were sealed and that there was no visible leakage from the strip. The MWA (maintenance work area) was prepped by setting up the miniPCR, ISS Station Support Computer (SSC) laptop, inverter (to change power from 120 VDC to 120 VAC), and the cables being connected. The sample and thermal cycling protocols were loaded onto the miniPCR machine, and the sample run was started. The astronaut allowed miniPCR operations to complete while working on other tasks, and the ground team monitored the miniPCR process via live video stream. Upon completion of the miniPCR run, the samples were allowed to cool for a minimum of 30 minutes before being removed from the miniPCR. Photos were captured to show the strip configuration prior to being placed back into the flight bag in the MELFI or GLACIER freezer, where they remained until loaded into the SpaceX Dragon inside a POLAR freezer set to -95°C. Upon splashing down to Earth in the Pacific Ocean off the coast of California, the samples were unloaded from the Dragon vehicle and placed in a freezer on a boat that took them to land. The samples were then placed inside a freezer for return to the Johnson Space Center, where the Boeing team packaged and sent them to Harvard University for analysis.

### Primer sequences

All semi-quantitative PCR primers were designed with one primer across a splice junction to avoid amplification of contaminating genomic DNA. The *act-1* primer sequences were previously published [26]. For primer sequences, see S1 Table.

### Gel band quantification

Quantification of DNA bands on raw gel image files (S1 and S2 Figs) was performed in FIJI (ImageJ) by drawing a rectangular region-of-interest within each band and calculating the mean grey value of that region. The relative grey value was calculated by dividing the no-heat shock value with the heat shock value for each condition.

## Results

### DNA extraction from *C*. *elegans* on board the ISS

**Fig 1:**
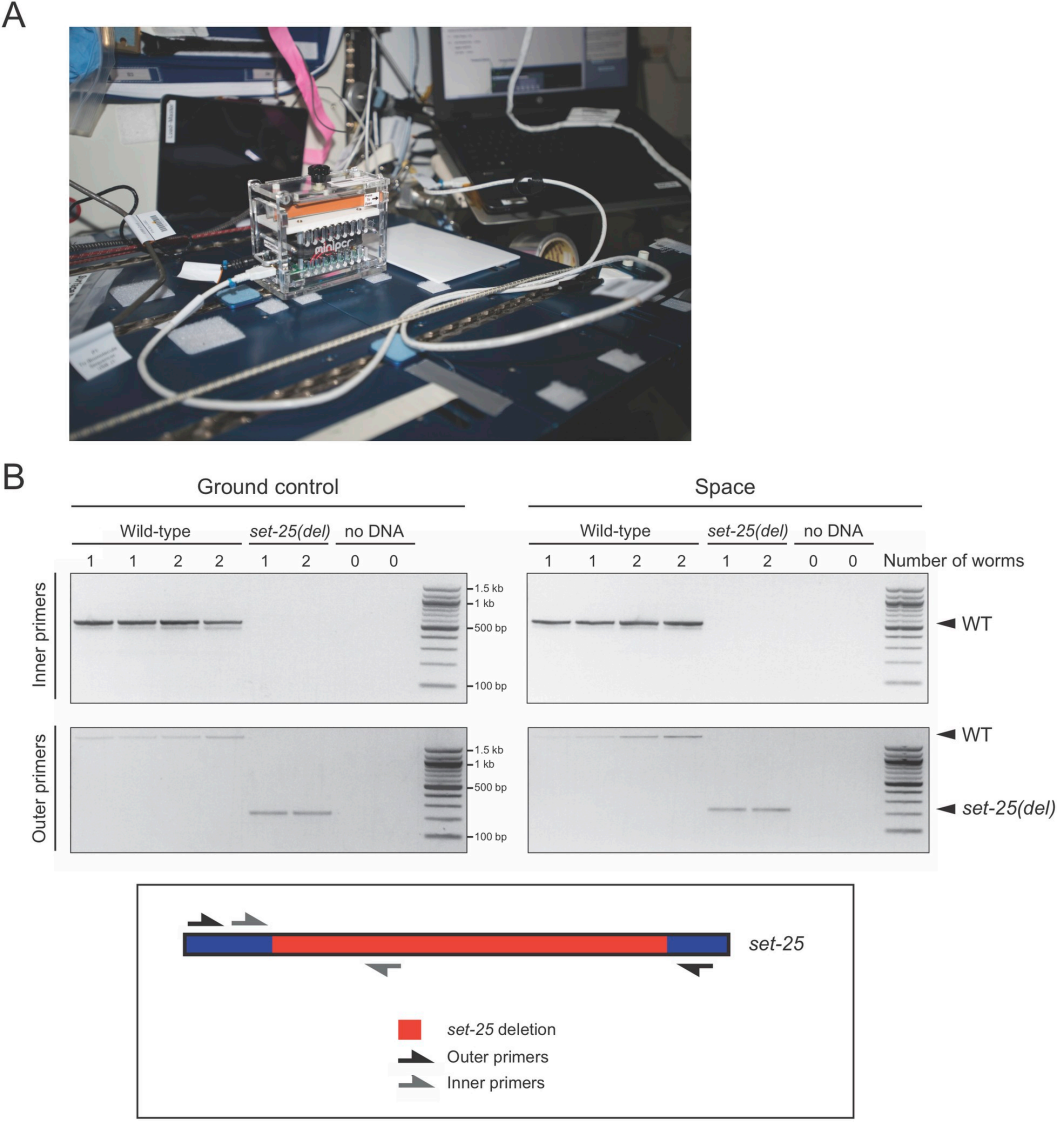
DNA extraction from *C*. *elegans* on board the ISS. (A) The miniPCR thermal cycler used during the Genes in Space-4 was set up in the maintenance work area and programmed and monitored through the onboard laptop computer (right on the background). NASA image in the public domain. (B) Either 1 or 2 individual wild-type and *set-25(del) C*. *elegans* were digested in lysis buffer and proteinase K on board the ISS in a miniPCR thermal cycler, as well as on Earth as a control. The resulting DNA was used as input in a PCR amplification reaction on Earth that tested for the presence of the deletion found in the *set-25* mutants. The *set-25* inner primers are designed to amplify a 573 bp product with wild-type DNA, but no product with *set-25* mutant DNA due to the deletion. The *set-25* outer primers are designed to amplify a 2220 bp band from WT animals, and a 242 bp band from *set-25* mutant animals.

To detect changes in an organism’s DNA, for instance any mutations induced by cosmic radiation, it is first necessary to extract DNA from biological tissues. To test the feasibility of DNA extraction in space we used *C*. *elegans* from two different genotypes: wild-type and a *set-25(n5021)* deletion strain [25]. Samples containing either 1 or 2 individual worms were mixed with lysis buffer and frozen, then half the samples were sent to the ISS while the other half remained on Earth. On board the ISS (and in parallel on Earth) the samples were incubated in a miniPCR thermal cycler (Fig 1A) at 60°C for 60 minutes followed by 95°C for 15 minutes to inactivate the proteinase K present in the buffer. The ISS samples were refrozen and sent back to Earth, where the genomic DNA from both conditions was tested in parallel as a template for amplification using the polymerase chain reaction (PCR). Two sets of primers were used: one flanking the *set-25(n5021)* deletion and another with one primer inside the deletion. In combination, these amplification reactions permitted the identification of the molecular lesion in the *set-25* mutants. Amplification from the extracted genomic DNA was successful, and the molecular lesion was identifiable in both the ground controls and ISS samples (Fig 1B), indicating that DNA can be successfully extracted in space with the same protocol as on Earth. Furthermore, our experiments reveal that DNA extracted on board the ISS can be frozen, stowed, and later utilized in molecular analyses back on Earth. Given the small volume used for DNA extraction, in future experiments, PCR reagents could be added directly to the DNA extraction reaction tubes to efficiently perform the complete DNA extraction and PCR in space.

### Semi-quantitative PCR in space

**Fig 2:**
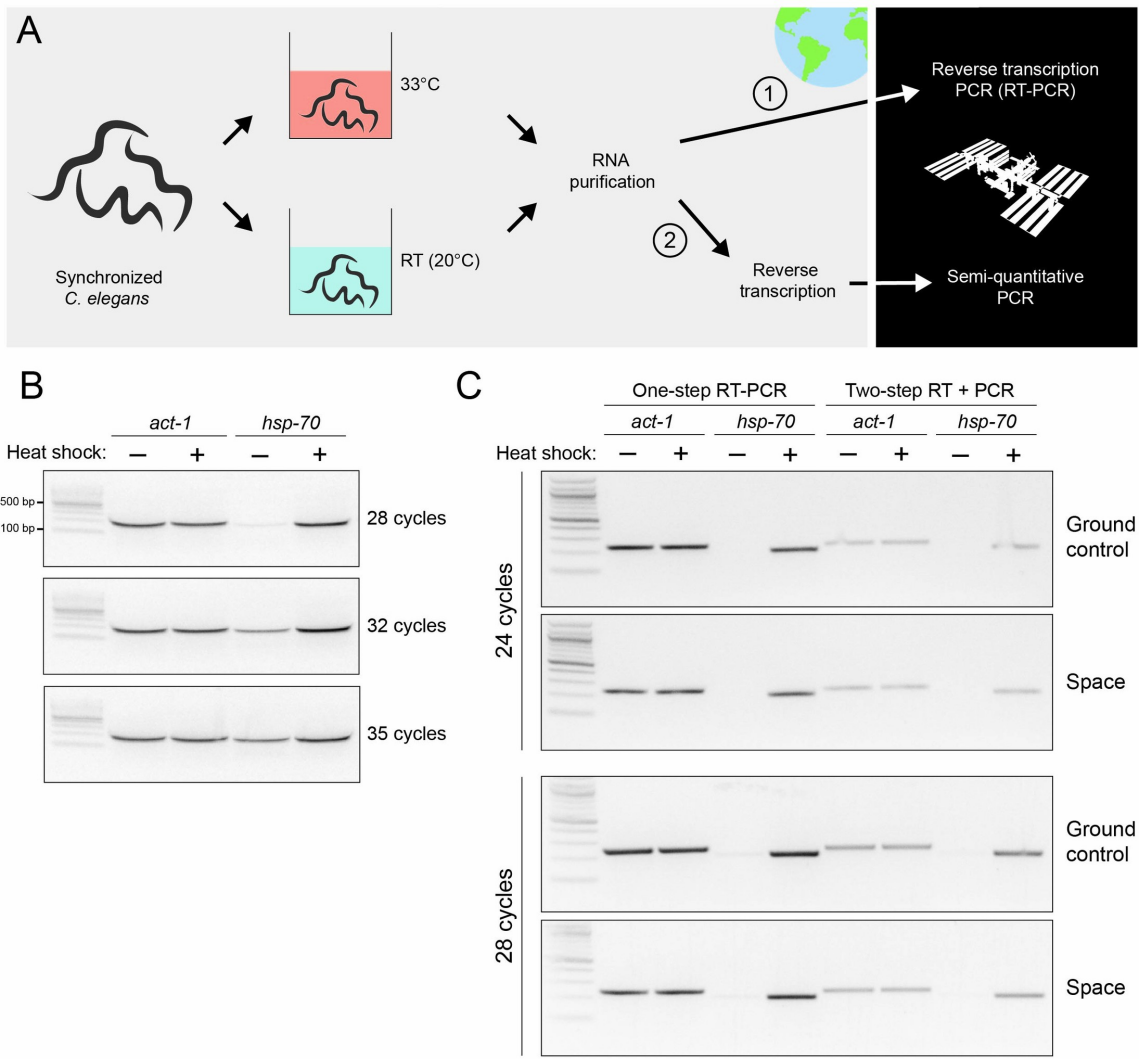
One-step reverse transcription and semi-quantitative DNA in space. (A) *C*. *elegans* were heat shocked for 30 minutes at 33°C to simulate space-induced changes in the expression of the heat shock gene *hsp-70*. Control worms were maintained at 20°C. After a 30-minute recovery period, RNA was purified from both populations of worms, and the RNA was subjected to DNase digestion. One set of samples was combined with reagents for one-step reverse transcription-PCR (RT-PCR) and frozen (1), while a second set of RNA samples was used as the input for a reverse transcription experiment on Earth, and the resulting cDNA was combined with PCR reagents and frozen (2). Half of each sample set was sent to the ISS while the other half remained on Earth as the ground control. Semi-quantitative PCR reactions were run on Earth and in space. All samples were analyzed on Earth using gel electrophoresis. (B) To determine the range of exponential DNA amplification of the *hsp-70* gene amplicon during PCR, cDNA was amplified using 28, 32 or 35 cycles of PCR. (C) Purified RNA samples were subjected to either RT-PCR, or reverse transcription followed by PCR. The samples were run on Earth and in space using 24 and 28 cycles of amplification. cDNA was amplified using primers targeting *hsp-70* to semi-quantitatively determine the amount of *hsp-70* gene expression in samples subjected to heat shock or no-heat shock. Amplification of *actin* (*act-1*) was used as a control. For raw gel images, see S1 and S2 Figs. Raw and relative grey values of gel bands in S1 and S2 Figs were quantified across heat shock conditions (S2 Table).

Due to limitations on the collection of live biological samples on board the ISS, we simulated space-induced changes in gene expression using the heat shock system of proteins in *C*. *elegans*. The heat shock proteins are part of a stress response system that is highly conserved from bacteria to humans [27]. Stresses such as heat or microgravity [28,29] cause up-regulation of the heat shock genes and synthesis of the heat shock proteins, which act as molecular chaperones to aid the correct folding of proteins [27]. We simulated spaceflight-induced expression of the *hsp-70* gene by subjecting *C*. *elegans* to a 30-minute heat shock on Earth, followed by purification of RNA from heat shock-treated and control animals after a 30-minute recovery period (Fig 2A).

To measure gene expression, we reverse transcribed RNA to generate cDNA, and then performed semi-quantitative DNA amplification. Semi-quantitative PCR uses a cycle number that lies within the exponential phase of DNA amplification so that the amount of product synthesized between different conditions is proportional to the amount of starting template. Primers are selected that target the gene of interest as well as a housekeeping (control) gene that is not expected to change between experimental conditions. We used primers targeting the heat shock gene *hsp-70* and the housekeeping gene *actin (act-1)*. We converted total RNA to cDNA on Earth, and then prepared two sets of reaction tubes containing cDNA and the PCR reagents. One set was stored at -80°C on Earth, and the other was sent frozen to the ISS. Astronaut Peggy Whitson performed semi-quantitative PCR in space using the protocol optimized on ground samples, while semi-quantitative PCR was performed in parallel on the ground controls. The ISS samples were then refrozen and returned to ground, where the results were analyzed in parallel by gel electrophoresis. During optimization of the procedure, parallel PCR experiments were run with different cycle numbers to determine when *hsp-70* amplification remains in the exponential phase (Fig 2B). This allowed for maximum distinction between the experimental conditions tested.

In space, the *act-1* product was amplified to equivalent levels under heat shock and no-heat shock conditions, similar to the ground controls (Fig 2C). The *hsp-70* product was undetectable, or almost undetectable in the no-heat shock condition, indicating that basal expression of *hsp-70* is low under normal physiological conditions. By contrast, under heat shock conditions, the *hsp-70* PCR product was amplified to high levels, indicating robust induction of *hsp-70* gene expression (Fig 2C). The mean grey values of each band, and the relative values across heat shock conditions, are provided in S2 Table. Thus, semi-quantitative PCR can be carried out in space to measure changes in gene expression, such as up-regulation of *hsp-70* triggered by stress conditions.

### One-step reverse transcription and semi-quantitative PCR in space

For molecular assays to be performed successfully by astronauts with limited resources and laboratory training, the workflow of sample collection, RNA extraction, reverse transcription and quantitative or semi-quantitative PCR should be as streamlined as possible. As a step towards a simplified procedure, we tested if one-step reverse transcription and semi-quantitative PCR in the same reaction tube, and without astronaut intervention, was feasible in space. We combined the RNA extracted on Earth with a complete enzyme mix, containing all of the reagents for reverse transcription and PCR in one tube. The samples were frozen and divided into two sets–one set was sent to space, where a combined reverse transcription-PCR (RT-PCR) protocol was performed on a miniPCR thermal cycler (Fig 2A). The second set was run in parallel on Earth. The RT-PCR successfully amplified the *act-1* and *hsp-70* amplicons in the ISS samples and ground controls, and produced similar results as the two-step reverse transcription and PCR (Fig 2C). Thus, using RNA as starting material, gene expression can be assessed with minimal astronaut intervention.

## Discussion

In this study we describe the successful execution of DNA extraction, reverse transcription and semi-quantitative PCR in space. Current ISS-based experiments generally rely on in-space sample collection followed by on-Earth processing and analysis. Our experiments reveal that three additional molecular techniques can be performed in space, thus expanding the molecular capabilities of the ISS. We show that these techniques can be conducted entirely in a miniaturized thermocycler system, which reduces time, space, cost of operating multiple pieces of equipment and training required for astronauts to perform numerous molecular biology procedures. Soon, astronauts will be able to generate and analyze data on their health and the molecular status of the living environment entirely in space.

In our study, we extracted DNA from nematode worms using a lysis buffer and heat. Extraction protocols that use an alkaline buffer and heat are commonly used to extract DNA from other model systems, such as mice and zebrafish [30,31]. Since these protocols also involve a simple heating and cooling step, it is likely that these procedures could also be adapted for use on the ISS to carry out DNA analysis in space. At the time of our study, exposing the contents of the tubes to the ISS environment was not feasible. However, as additional biological containment methods are added to the ISS, and astronauts become familiar with pipetting and other basic molecular biology techniques, scientists will have more flexibility in the types of DNA extractions and subsequent reactions that can be carried out in space. For example, our findings could aid scientists and astronauts in developing sample preparation procedures for next generation sequencing and other downstream DNA analyses.

One shortcoming of our study was that the enzyme inactivation step in the DNA extraction protocol was carried out for 1.5 minutes in space instead of 15 minutes. This step normally ensures that any proteinase K transferred with template DNA into the PCR reaction does not inactivate the polymerase. To overcome this issue, we completed the enzyme inactivation step upon the return of the samples to Earth. This did not appear to affect the amplification of DNA, as we achieved robust amplification from these samples (Fig 1B). It is possible that the equivalent heating step would not have achieved the same effect in space, but this is unlikely, given that PCR reactions (which include similar heating steps) were successfully completed in space (Fig 2C).

A round-trip mission to Mars is expected to take approximately 3 years [32]. The astronauts embarking on this journey will need to be entirely self-sufficient to survive the hostile environment of deep space. Not only will they be solely responsible for their own health, but upon exiting Earth’s magnetosphere, the astronauts will be subject to approximately three times the cosmic radiation as spacecraft within Earth’s orbit [32]. In order to closely monitor their health, crewmembers will need to track not only their physiology, but also their DNA integrity. By successfully demonstrating DNA extraction in space, we are one step closer to astronauts acquiring their own biological samples, extracting the DNA and sequencing it, for example using the MinION nanopore sequencer that has been successfully operated in microgravity [20]. In addition, if a crewmember acquires an infection, in the future, astronauts could theoretically use DNA analysis to identify the organism and any genetic changes it has undergone to inform their decisions for treatment.

The human body has been shown to undergo gene expression changes in space, which could reveal underlying health issues or changes to physiology, such as cellular stress or inflammation. By demonstrating successful reverse transcription of RNA and semi-quantitative PCR on board the ISS, we provide evidence that two of the critical steps of gene expression analysis can be carried out in space. Future developments should take us closer to the ultimate goal of allowing astronauts to carry out full genetic and gene expression analyses in space using human samples.

## Supporting information

S1 Fig Raw DNA gel from Fig 2C (24 cycles). (TIF)

S2 Fig Raw DNA gel from Fig 2C (28 cycles). (TIF)

S1 Table PCR primer sequences. (DOCX)

S2 Table Quantification of DNA gel bands in S1 and S2 Figs (unprocessed versions of Fig 2C). (DOCX)
