# Supplementary figures and images for: Gene expression studies using a miniaturized thermal cycler system on board the International Space Station
Source: PLoS One. 2018 Oct 31;13(10):e0205852. doi: 10.1371/journal.pone.0205852 (PMC6209215; doi:10.1371/journal.pone.0205852)

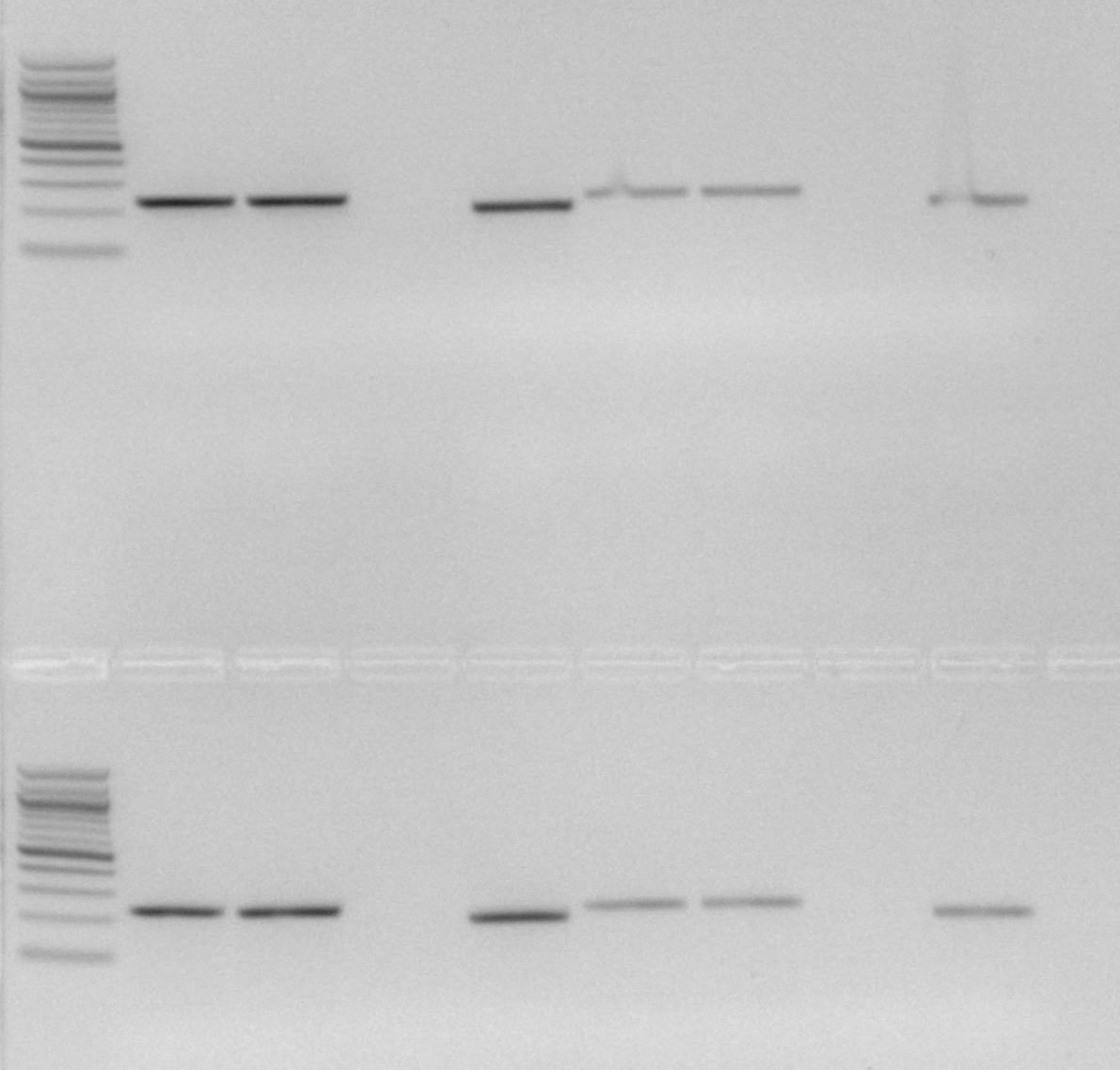

Supplement: S1 Fig — (TIF) [file pone.0205852.s001.tif]

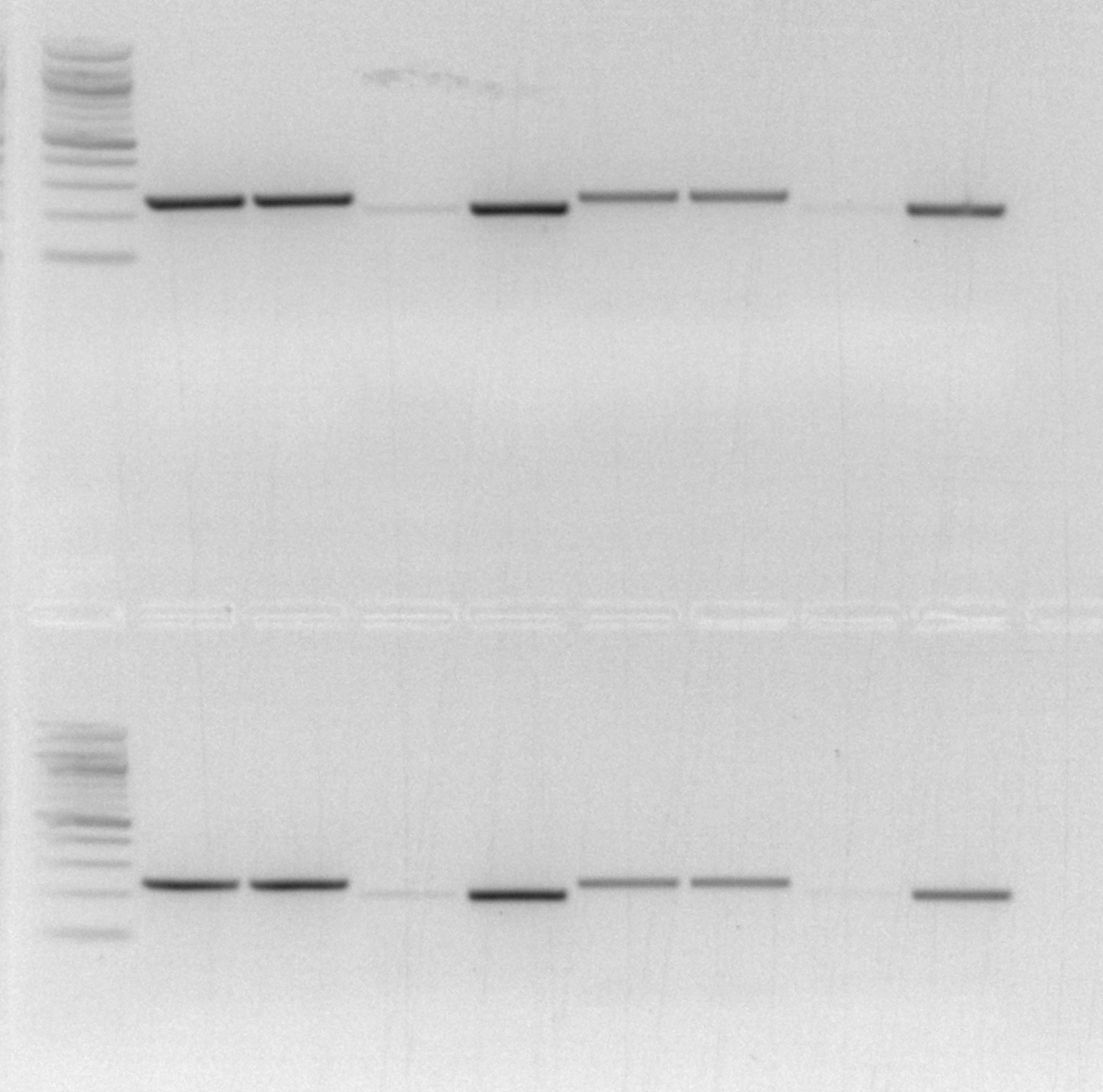

Supplement: S2 Fig — (TIF) [file pone.0205852.s002.tif]
