# Supplementary material for: Gene expression studies using a miniaturized thermal cycler system on board the International Space Station
Source: PLoS One. 2018 Oct 31;13(10):e0205852. doi: 10.1371/journal.pone.0205852 (PMC6209215; doi:10.1371/journal.pone.0205852)
Supplement: S1 Table — (DOCX) [file pone.0205852.s003.docx]

| Set-25_outer_F | TTCGTTGGTTTTTCGGACA |
| --- | --- |
| Set-25_outer_R | CCGTGCTACGCGGTAAGTAT |
| Set-25_inner_F | AGACTTCGACGAACACCGAG |
| Set-25_inner_R | CGCGTGGAAAAGTTTCGTGT |
| Act-1_F | AATCCAAGAGAGGTATCCTTA |
| Act-1_R | GATGGCGACATACATGGCT |
| Hsp-70_F | GAAAATCACACGTGCAAGATTCG |
| Hsp-70_R | GAGCAGTTGAGGTCCTTCCC |

**Table S1.** **PCR primer sequences.**
