# Supplementary material for: Gene expression studies using a miniaturized thermal cycler system on board the International Space Station
Source: PLoS One. 2018 Oct 31;13(10):e0205852. doi: 10.1371/journal.pone.0205852 (PMC6209215; doi:10.1371/journal.pone.0205852)
Supplement: S2 Table — (DOCX) [file pone.0205852.s004.docx]

| **PCR type** | **Cycle number** | **Location** | **Primer** | **Heatshock (HS)** | **Mean intensity of band (grey value)** | **Relative grey values (-HS / +HS)** |
| --- | --- | --- | --- | --- | --- | --- |
| One-step RT-PCR | 24 | Earth | act-1 | - | 25.628 | 1.03 |
|  |  |  |  | + | 24.977 |  |
|  |  |  | hsp-70 | - | 187.13 | 3.48 |
|  |  |  |  | + | 53.742 |  |
|  |  | Space | act-1 | - | 56.739 | 1.00 |
|  |  |  |  | + | 56.612 |  |
|  |  |  | hsp-70 | - | 194.152 | 3.17 |
|  |  |  |  | + | 61.173 |  |
|  | 28 | Earth | act-1 | - | 41.964 | 1.09 |
|  |  |  |  | + | 38.428 |  |
|  |  |  | hsp-70 | - | 178.094 | 9.85 |
|  |  |  |  | + | 18.086 |  |
|  |  | Space | act-1 | - | 53.793 | 1.25 |
|  |  |  |  | + | 42.91 |  |
|  |  |  | hsp-70 | - | 188.647 | 5.80 |
|  |  |  |  | + | 32.549 |  |
| 2-step RT + PCR | 24 | Earth | act-1 | - | 138.521 | 1.02 |
|  |  |  |  | + | 136.372 |  |
|  |  |  | hsp-70 | - | 188.072 | 1.49 |
|  |  |  |  | + | 126.507 |  |
|  |  | Space | act-1 | - | 138.644 | 0.94 |
|  |  |  |  | + | 147.592 |  |
|  |  |  | hsp-70 | - | 197.094 | 1.41 |
|  |  |  |  | + | 139.526 |  |
|  | 28 | Earth | act-1 | - | 104.358 | 0.98 |
|  |  |  |  | + | 106.467 |  |
|  |  |  | hsp-70 | - | 189.707 | 2.47 |
|  |  |  |  | + | 76.724 |  |
|  |  | Space | act-1 | - | 121.907 | 0.99 |
|  |  |  |  | + | 123.392 |  |
|  |  |  | hsp-70 | - | 202.584 | 2.00 |
|  |  |  |  | + | 101.467 |  |

**Table S4. Quantification of DNA gel bands in Figs S2 and S3 (unprocessed versions of Fig 2C).**
